# On the Local Application of the Vapor of Chloroform

**Published:** 1854-02

**Authors:** 


On the Local Application of the Vapor of Chloroform.—In an article in the *Dublin Quarterly Journal*, Dr. Hardy gives several examples of the application of the vapor of chloroform to the vagina and neck of the uterus, in painful affections of these parts, by means of an instrument which he has invented for that purpose. The following are his conclusions:—

"In observing the effects of chloroform as applied locally in the form of vapor in the above cases, I have endeavored to obtain as correct a notion of it as possible, in order that a true estimate might be arrived at of its value as a remedy. Besides the cases here recorded, I have applied the vapor locally in various other forms of irritation. One of these in particular, I was anxious to know its action in—namely, *pruritus pudendi*, a disease exceedingly troublesome and unpleasant to the patient, and for the relief of which she is often very reluctant to ask a remedy until forced to do so. I have used it in a case of this kind in the person of a very intelligent patient, who for a length of time had been annoyed, particularly on the approach of a menstrual period, by this distressing complaint, for which she had made use of various remedies. The vapor of chloroform, she informed me, afforded her relief from her uneasy sensations. On referring to one of the cases (Case v.) detailed, it will be seen that there was a very severe sense of scalding in the vagina, which seemed to depend a good deal on uterine irritation. Knowing the heat caused by the vapor of chloroform, I feared this patient should have suffered severely from its application; but, on the contrary, she was quite relieved of it; so in *pruritus pudendi*, arising from a similar cause, the like results have been obtained as in her case.

"If future investigations as to the effect of the vapor of chloroform when locally applied coincide with the results already observed in the series of cases herein detailed, it seems reasonable that the following conclusions be considered deducible:—

"*First.* That in many forms of disease attended with pain or irritation, the local application of the vapor of chloroform will frequently act as quickly in affording immunity from suffering as though inhaled in the usual manner.

"*Secondly.* That the vapor locally applied is not attended with any unpleasant effects (save the sensation of more or less heat) either at the time or subsequently, and is therefore eligible under circumstances contra-indicating its use by inhalation.

"*Thirdly.* That as a remedy its local application is preferable to the use of opium and most narcotics in spasmodic and painful affections, particularly of the uterine system, owing, first, to its freedom from causing derangement of the digestive organs, and, secondly, to its greater rapidity of action."—*N. Y. Jour. Med.*

